# Extracellular Vesicles Induce Nuclear Factor-κB Activation and Interleukin-8 Synthesis through miRNA-191-5p Contributing to Inflammatory Processes: Potential Implications in the Pathogenesis of Chronic Obstructive Pulmonary Disease

**DOI:** 10.3390/biom14081030

**Published:** 2024-08-19

**Authors:** Sara Carpi, Beatrice Polini, Dario Nieri, Stefano Doccini, Maria Conti, Erika Bazzan, Marta Pagnini, Filippo Maria Santorelli, Marco Cecchini, Paola Nieri, Alessandro Celi, Tommaso Neri

**Affiliations:** 1 Department of Health Sciences, University ‘Magna Græcia’ of Catanzaro, 88100 Catanzaro, Italy; sara.carpi@unicz.it; 2 NEST, Istituto Nanoscienze-CNR and Scuola Normale Superiore, Piazza San Silvestro 12, 56127 Pisa, Italy; marco.cecchini@nano.cnr.it; 3 Department of Pathology, University of Pisa, 56100 Pisa, Italy; beatrix0087@gmail.com; 4 Centre for Cardio-Respiratory Cell Biology, Department of Surgical, Medical and Molecular Pathology and Critical Care Medicine, University of Pisa, 56100 Pisa, Italy; darionieri@hotmail.it (D.N.); marta.pagnini@med.unipi.it (M.P.); tommaso.neri@unipi.it (T.N.); 5 Molecular Medicine for Neurodegenerative and Neuromuscular Diseases Unit, IRCCS Stella Maris Foundation, 56128 Pisa, Italy; stefano.doccini@fsm.unipi.it (S.D.); filippo3364@gmail.com (F.M.S.); 6 Department of Cardiac, Thoracic, Vascular Sciences and Public Health, University of Padova, 35122 Padua, Italy; maria.conti.2@phd.unipd.it (M.C.); erica.bazzan@unipd.it (E.B.); 7 Department of Pharmacy, University of Pisa, 56100 Pisa, Italy; paola.nieri@unipi.it

**Keywords:** extracellular vesicles, chronic obstructive pulmonary disease, microRNA-191, inflammation, nuclear factor-κB, interleukin-8, interleukin-6, DLCO

## Abstract

Extracellular vesicles (EVs) play a pivotal role in a variety of physiologically relevant processes, including lung inflammation. Recent attention has been directed toward EV-derived microRNAs (miRNAs), such as miR-191-5p, particularly in the context of inflammation. Here, we investigated the impact of miR-191-5p-enriched EVs on the activation of NF-κB and the expression of molecules associated with inflammation such as interleukin-8 (IL-8). To this aim, cells of bronchial epithelial origin, 16HBE, were transfected with miR-191-5p mimic and inhibitor and subsequently subjected to stimulations to generate EVs. Then, bronchial epithelial cells were exposed to the obtained EVs to evaluate the activation of NF-κB and IL-8 levels. Additionally, we conducted a preliminary investigation to analyze the expression profiles of miR-191-5p in EVs isolated from the plasma of patients diagnosed with chronic obstructive pulmonary disease (COPD). Our initial findings revealed two significant observations. First, the exposure of bronchial epithelial cells to miR-191-5p-enriched EVs activated the NF-kB signaling and increased the synthesis of IL-8. Second, we discovered the presence of miR-191-5p in peripheral blood-derived EVs from COPD patients and noted a correlation between miR-191-5p levels and inflammatory and functional parameters. Collectively, these data corroborate and further expand the proinflammatory role of EVs, with a specific emphasis on miR-191-5p as a key cargo involved in this process. Consequently, we propose a model in which miR-191-5p, carried by EVs, plays a role in airway inflammation and may contribute to the pathogenesis of COPD.

## 1. Introduction

Chronic obstructive pulmonary disease (COPD) is a heterogeneous condition defined by the presence of chronic respiratory symptoms and largely irreversible airflow limitation [1], ranking as the third leading cause of mortality worldwide [2]. Both pulmonary and systemic inflammation play a pivotal role in COPD pathogenesis, but the exact pathologic mechanisms underlying the disease remain incompletely understood [3]. Current therapeutic approaches primarily rely on β_2_-agonists, muscarinic antagonists, and steroids, which improve patients’ quality of life, reduce acute exacerbations (defined as sudden worsening of respiratory symptoms sometimes requiring hospitalization) [4], and potentially enhance overall survival [5]. Nonetheless, a more comprehensive understanding of the disease mechanisms is imperative for the development of novel therapeutic strategies [6].

Cells release vesicles either constitutively, upon stimulation, or during apoptosis. At least three different categories of cell-derived vesicles have been described, namely exosomes, microparticles, and apoptotic bodies [7]. While these structures differ in terms of biogenesis, the distinction among these vesicle types in most experimental contexts remains challenging, and the International Society for Extracellular Vesicles endorses the use of the generic term “extracellular vesicles” (EVs) [8]. EVs have been implicated in a variety of physiologically relevant processes, including blood coagulation and inflammation [7]. We have previously shown that EVs derived from mononuclear cells exposed to different stimuli upregulate the synthesis of interleukin (IL)-8, a key regulator of inflammation in COPD [9], in pulmonary epithelial cells [10] through the activation of the nuclear transcription factor, nuclear factor kappa-light-chain-enhancer of activated B cells (NF-κB) [11]. We have also demonstrated that the adhesion of EVs to epithelial cells, mediated by cluster of differentiation (CD)-18, is a prerequisite for this effect [12].

EVs have generated considerable attention in recent years in a variety of clinically relevant contexts, including COPD, where EVs have been investigated essentially as possible biomarkers of several aspects of the disease [13]. However, their role as disease mediators, and therefore as potential therapeutic targets, has not yet been established. We have generated data that indicate that EVs isolated from the peripheral blood of COPD patients during an acute exacerbation stimulate IL-8 synthesis by epithelial cells to a greater extent compared with EVs collected 8 weeks after the acute event. This result is compatible with a direct pathogenic role for EVs in COPD [14].

EVs transport a variety of cargo, including proteins, lipids, and DNA and RNA sequences, including microRNAs (miRNAs). The latter are short, single-stranded RNA transcripts typically consisting of 20–22 nucleotides, capable of modulating the expression levels or translation rates of specific mRNA targets [15]. miRNAs are taken up by other cells, either close to the source or at distant sites, and in recipient cells trigger a variety of phenotypic responses. Indeed, a large body of evidence suggests that miRNA cargo in EVs can affect gene expression and function in recipient cells [16]. Numerous miRNAs have been investigated in the context of COPD [17,18], and among them, microRNA-191-5p (miR-191-5p) caught our interest. It is highly conserved in eukaryotes and extensively studied in various types of diseases and biological pathways, with a particular focus on inflammation [19]. miR-191-5p has been implicated in the activation of NF-κB signaling through the upregulation of subunit p65 in endothelial cells [20], and a positive correlation has been observed between miR-191 levels and markers of inflammation, such as C-reactive protein and proinflammatory cytokine levels [21]. Notably, circulating plasma miR-191 levels are higher in circulating endothelial microparticles of individuals exposed to cigarette smoke [22], as well as in smokers more than in non-smokers [23], and more abundant in subjects with pulmonary hypertension compared with healthy subjects [24].

To test the hypothesis that the activation of the NF-κB pathway mediated by EVs is, at least in part, mediated by the transfer of miR-191-5p to the target epithelial cells, we exposed bronchial epithelial cells to miR-191-5p-enriched EVs, evaluating NF-κB activation and IL-8 production. To further investigate the potential role of this pathway in the pathogenesis of COPD, we then assessed the expression levels of miR-191-5p in plasma-derived EVs from patients with COPD, examining their correlations with both IL-6 levels and clinical parameters.

Our findings highlight the role of miR-191-5p-containing EVs in promoting bronchial inflammation in vitro and show a significant relationship between circulating miR-191-5p and clinically relevant parameters in a cohort of COPD patients.

## 2. Materials and Methods

### 2.1. Study Design

The objective of this study was to investigate the impact of miR-191-5p-enriched EVs on the activation of NF-κB and the expression of inflammatory molecules, such as IL-8, in bronchial epithelial cells (16HBE). Additionally, we aimed to analyze the expression profiles of miR-191-5p in EVs isolated from the plasma of patients diagnosed with COPD and assess the correlations with clinical parameters.

In detail, 16HBE cells were transfected with miR-191-5p mimic, miR-191-5p inhibitor, and control using Lipofectamine 2000^®^. EVs were generated from Ach-stimulated 16HBE cells, a process we have thoroughly explored and detailed in a previous publication [25] and characterized. Simultaneously, a clinical study involving COPD patients in a stable phase of the disease was conducted. EVs were isolated from the plasma of COPD patients.

**Figure 1:**
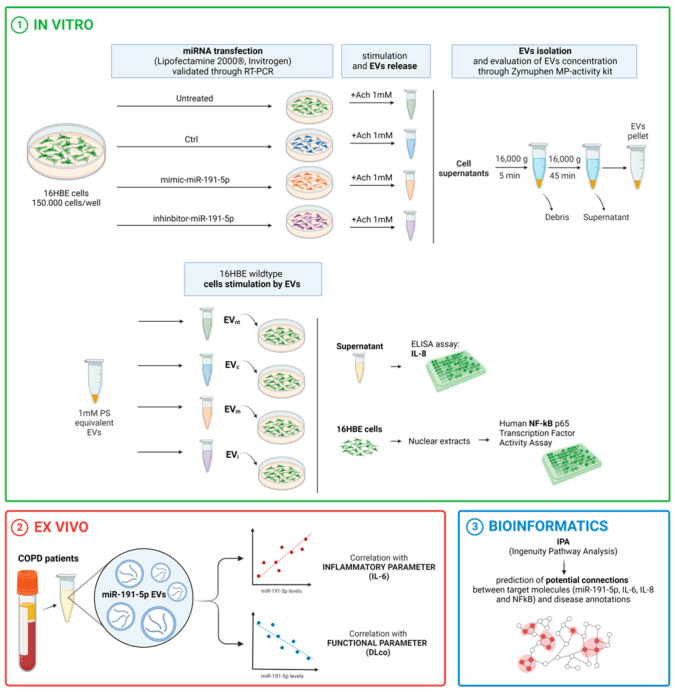
Graphical overview of the experimental design. Bronchial epithelial cells (16HBE) were transfected with miR-191-5p mimic or inhibitor. Subsequently, supernatants were collected for EV isolation and miR-191-5p quantification. Additionally, cells were harvested for miRNA extraction. The bronchial epithelial cells were then exposed to EVs (EV_m_: EV derived from 16HBE transfected with miR-191-5p mimic; EV_i_: EV derived from 16HBE transfected with miR-191-5p inhibitor; EV_c_: EV derived from 16HBE transfected with miRNA scramble; EV_nt_: EV derived from 16HBE not transfected) and subsequently collected for miR-191-5p quantification, NF-kκb activation and IL-8 evaluation. The figure was created through “biorender.com” website license agreement number EX2679WEP4, access date 11 December 2024.

miRNAs were extracted and purified from 16HBE cells, and EVs were isolated from cell medium and from plasma of COPD patients using the miRNeasy Micro Kit. miR-191-5p expression was assessed using qPCR and normalized to reference (Cel-miR-39 and SNORD38B). IL-6 concentrations were measured in plasma using a sandwich ELISA kit. IL-8 levels in cell culture supernatants were measured using ELISA kits. After EV exposure, nuclear extracts were obtained from 16HBE cells. NF-kB activation was evaluated in nuclear extracts using a transcription factor activity assay. Ingenuity pathway analysis (IPA) was used for network analysis and prediction of potential connections between target molecules and disease annotations (Figure 1 study design). The endpoints of the studies were (i) measurement of miR-191-5p levels in EVs and recipient cells, (ii) evaluation of NF-κB activation and IL-8 production in bronchial epithelial cells, and (iii) correlation of EV-derived miR-191-5p levels with clinical parameters in COPD patients.

### 2.2. Cell Culture

Immortalized bronchial epithelial cells (16HBE) (American Type Culture Collection, CRL-2741) were kindly provided by Dr. M. Profita (National Research Council, Palermo, Italy). 16HBE cells were maintained in MEM supplemented with 10% (vol/vol) FBS, 0.2 mL/mL L-glutamine, and 2.5 mM HEPES buffer as described (Merk, Milan, Italy) [12]. Cell line was maintained in a humidified 95% air/5% CO_2_ atmosphere at 37 °C.

### 2.3. Cell Transfection with miR-191-5p Mimic and miR-191-5p Inhibitor

miRNA mimic of miR-191-5p (m-miR-191-5p) and miR-191-5p (i-miR-191-5p) inhibitor were purchased from Qiagen (Hilden, Germany). The mimic scramble was used as negative control (Ctrl) in each experiment. Cell lines were plated at 60% confluence and transfected with 20 nM mimic (m-miR-191-5p or i-miR-191-5p inhibitor) or Ctrl using Lipofectamine 2000^®^ (Invitrogen, Thermo Fisher Scientific, Waltham, MA, USA), according to the manufacturer’s protocol. Non-transfected cells represent the not-treated group (Untreated).

### 2.4. Evaluation of miR-191-5p Expression

MiRNAs were extracted and purified from 16HBE cells, and EVs were isolated from cell medium and plasma of COPD patients using the miRNeasy Micro Kit (Qiagen, Hilden, Germany). MiRNAs were retro-transcribed, and qPCR experiments were conducted as previously reported [18]. A MiScript Primer Assay specific to hsa-miR-191-5p (MIMAT0000440, sequence: 16-CAACGGAAUCCCAAAAGCAGCUG-38) was used. MiR-191-5p expression was calculated using the Delta threshold cycle (Ct) method and normalized to *Caenorhabditis elegans* miR-39 (Cel-miR-39) and SNORD38B for miRNA derived from EVs or cells, respectively.

### 2.5. Elisa for IL-6 and IL-8 Detection

Plasma concentrations of IL-6 were measured by a sandwich ELISA kit (Kit- Elisa-Ready-SET-Go!, Affymetrix, Santa Clara, CA, USA) with a microplate reader (iMarkTM Microplate Absorbance Reader, Bio-Rad, Milan, Italy), according to the manufacturer’s instructions. IL-8 in supernatants from 16HBE cells was measured by sandwich ELISA kits (Biosource International, San Diego, CA, USA), according to the manufacturer’s instructions.

### 2.6. NF-kB Activity Evaluation

After challenge with EVs, 16HBE cells were washed with ice-cold PBS, scraped, and centrifuged at 1000× *g* for 5 min at 4 °C. Nuclear extracts were obtained with a commercially available kit (NE-PER Nuclear and Cytoplasmatic Extraction Reagents, Thermo Fisher Scientific, USA), according to the manufacturer’s instructions.

NF-kB was evaluated in nuclear extract of 16HBE cells by Human NF-KappaB p65 Transcription Factor Activity Assay (RayBiotech, Peachtree Corners, GA, USA), according to the manufacturer’s instructions.

### 2.7. Study Cohort and Functional Tests

The clinical study was approved by the local ethics committee (approval code n. 1088, 15 June 2016) and was carried out in compliance with the Declaration of Helsinki. All subjects signed written informed consent forms. The study population has been previously published [18].

Briefly, we enrolled patients with COPD, diagnosed according to current indications (post-bronchodilator forced expiratory volume in the first second (FEV1) to forced vital capacity ratio < 0.7 and clinical history [1], with FEV1 between 30% and 70% of the predicted value). Patients with a history of asthma and with major comorbidities such as recent acute cardiovascular events and active cancer) were excluded. All patients were free of acute exacerbations within the last 6 weeks.

All patients underwent a complete functional characterization, including spirometry, measurement of lung volumes, and diffusion lung capacity for carbon monoxide (DLCO), according to current guidelines [26,27,28]. In COPD, DLCO essentially correlates with the extent of pulmonary emphysema; in less frequent cases, it can also provide information on the presence of pulmonary hypertension as a result of vascular involvement and chronic hypoxia [1,29].

### 2.8. Isolation and Characterization of Extracellular Vesicles

#### 2.8.1. From Plasma of Patients

Circulating EVs were obtained from 4 mL of peripheral blood. Briefly, platelet-poor plasma (PPP) was prepared through two consecutive centrifugations: first at 1500× *g* for 15 min, followed by 13,000× *g* for 2 min, both at room temperature. The PPP was then stored at −80 °C until it was utilized for EV analysis. For the analysis of miRNA contained in EVs, we isolated EVs from platelet-poor plasma. Briefly, blood was drawn into sodium citrate. Platelet-poor plasma (PPP) was obtained by two subsequent centrifugations: 1500× *g* for 15 min, and 13,000× *g* for 2 min at room temperature. PPP was then centrifuged at 16,000× *g* for 45 min to obtain EV pellets. EV pellets were stored at −80 °C and subsequently used for EV analysis.

#### 2.8.2. From Cell Medium

Immortalized bronchial epithelial (16HBE) cells, grown until 90% confluent, were washed twice with pre-warmed phosphate-buffered saline prior to stimulation. For EV generation, Ach was resuspended first in distilled water and then in cell medium and added at 1 mM. After 60 min at 37 °C, the supernatants were recovered and cleared by centrifugation at 16,000× *g* for 5 min at room temperature to remove cells and big cell fragments that might have detached during the stimulation. The free cells supernatant was then centrifuged at 16,000× *g* for 45 min to obtain EV pellets. EV concentrations were evaluated by Zymuphen MP-activity kit (Hyphen BioMed, Neuville-sur-Oise, France), according to the manufacturer’s instructions, and expressed as PS equivalents (nM PS). EVs used for all experiments were normalized to a value of 1 nM.

### 2.9. Network Analysis and Molecules Activity Prediction

Network analysis was carried out using the Ingenuity Pathway Analysis software platform (version: 111725566, release number: summer release, Q2 2024; QIAGEN, Hilden, Germany) [30]. Interaction networks were generated by selecting mutual interactions experimentally observed (maximum confidence) between target molecules (miR-191-5p, IL6, IL8, NF-kB) and significant disease annotations in the category of Respiratory Disease with predictable scores of activation and inhibition (Inflammation of respiratory system component; Inflammation of lung, Damage of lung, Fatigue). The *p*-value, which was ascertained by right-tailed Fisher’s Exact Test following Benjamini and Hochberg correction, indicates the robustness of correlations. The Path Explorer tool was used to identify the shortest molecular paths between molecules or between molecules and diseases of interest and to generate a hierarchical signaling pathway able to explain the consequence of target modulation. Moreover, the IPA algorithm estimated the predicted activation or inhibition of a given connecting node or biological function, simulating the directional consequences associated with the modulated miRNAs or mRNAs.

### 2.10. Statistical Analysis

In vitro experiments were carried out in technical triplicate and biological triplicate, as a minimum. To assess the reproducibility of the results, the coefficient of variation in both intra- and inter-experiments was evaluated, which never exceeded 10%. Graphic representation and statistical analysis of data were performed using GraphPad Prism 8.0 (GraphPad Software, San Diego, CA, USA). The data are shown as the mean value ± SD (standard deviation) or median (interquartile range) as appropriate, and box plot (10–90 percentile).

We performed multiple comparisons by one-way analysis of variance (ANOVA) with Tukey’s multiple comparison test. Pearson analysis was applied to investigate potential correlation between miR-191-5p levels and clinical parameters. A *p* level of <0.05 was considered statistically significant.

The dataset of experiments has been deposited to Zenodo’s open-access archive in order to preserve the research results.

## 3. Results

### 3.1. miR-191-5p-Enriched EV Increased miR-191-5p in Recipient Cells

**Figure 2:**
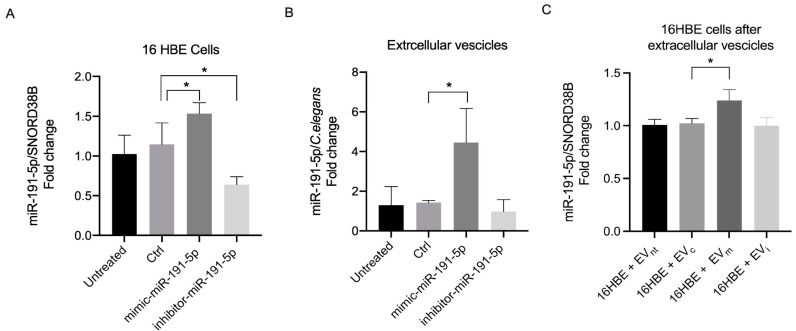
Expression of miR-191-5p in 16HBE cells (**A**) and in EVs (**B**) after transfection with miRNA mimic of miR-191-5p (mimic-miR-191-5p) and miR-191-5p inhibitor (inhibitor -miR-191-5p). The mimic scramble was used as control (Ctrl) in each experiment. Non-transfected cells represent the not-treated group (Untreated). Expression of miR-191-5p in 16HBE cells (**C**) after incubation with EVs shed from bronchial epithelial cells transfected with mimic-miR-191-5p and inhibitor-miR-191-5p (EV_m_: EV derived from 16HBE transfected with miR-191-5p mimic; EV_i_: EV derived from 16HBE transfected with miR-191-5p inhibitor; EV_c_: EV derived from 16HBE transfected with miRNA scramble; EV_nt_: EV derived from 16HBE not transfected). Statistical analysis was performed using one-way ANOVA followed by Bonferroni’s test selected data (* *p* < 0.05 vs. Ctrl).

miR-191-5p levels were increased in cells transfected with mimic and were reduced in cells transfected with the inhibitor compared with control cells, confirming successful transfections (Figure 2A). Furthermore, miR-191-5p levels were also increased in EVs derived from cells transfected with miR-191-5p mimic compared with controls. Conversely, no significant decrease was observed in EVs derived from cells transfected with miR-191-5p inhibitor, possibly indicating that the decrease in miR-191-5p in cells transfected with the inhibitor is not crucial to determine the extent of miR-191-5p loaded into EVs derived from stimulated cells (Figure 2B).

After obtaining miR-191-5p-enriched EVs, we tested whether these EVs induce a coherent increase in miR-191-5p levels within 16HBE recipient cells. We observed a statistically significant increase in miR-191-5p levels in recipient cells exposed to EVs derived from 16HBE transfected with miR-191-5p mimic (EV_m_) compared with controls (Figure 2C).

### 3.2. miR-191-5p-Enriched EVs Activate the Inflammatory Pathway

Given that the transcription factor NF-κB serves as a master regulator of inflammatory responses [31], we examined the impact of miR-191-5p-enriched EVs on the activation of NF-κB in human bronchial epithelial cells. To this end, 16HBE cells were exposed to EV_m_, EV_i_, EV_c_, and EV_nt_ for 1 h to evaluate NF-κB activation and nuclear translocation. We quantified the binding capacity of the p65 subunit of NF-κB to the DNA consensus site, which is positively correlated with NF-κB activation, in the nuclear extracts using an enzyme-linked immunosorbent assay (ELISA).

**Figure 3:**
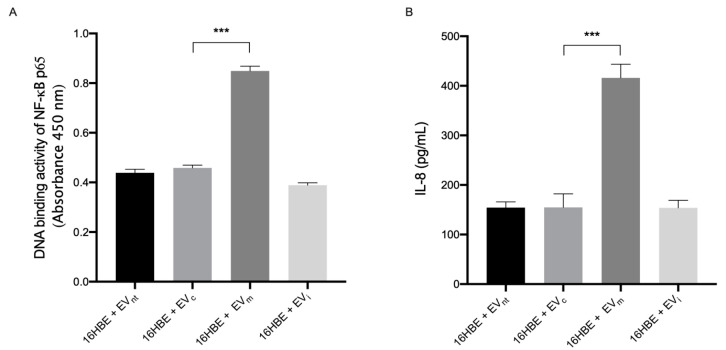
Modulation of NF-κB and IL-8 by miR-191-5p-enirched EVs. (**A**) Activation of NF-κB in 16HBE cells exposed to EV derived from Ach-stimulated 16HBE cells treated as described in Figure 3A (EV_m_: EV derived from 16HBE transfected with miR-191-5p mimic; EV_i_: EV derived from 16HBE transfected with miR-191-5p inhibitor; EV_c_: EV derived from 16HBE transfected with miRNA scramble; EV_nt_: EV derived from 16HBE not transfected). Then, nuclear proteins were extracted and assessed by ELISA to measure the DNA-binding activity of the p65 NF-κB subunit. (**B**) IL-8 secretion by 16HBE cells incubated with EVs derived from Ach-stimulated 16HBE cells, treated as described in Figure 3A. Statistical analysis was performed using one-way ANOVA followed by Tukey’s multiple comparison test (*** *p* < 0.001 vs. 16HBE + EV_c_).

As shown in Figure 3A, we detected a marked increase (approximately 100%) in the DNA-binding activity of the p65 NF-κB subunit in 16HBE cells exposed to EV_m_ compared with controls, underlining the activation of NF-κB in response to miR-191-5p.

Furthermore, 16HBE cells were incubated with EV_m_, EV_i_, EV_c_, and EV_nt_ for 18 h to assess the concentration of IL-8 in the supernatants (Figure 1 study design). As reported in Figure 3B, IL-8 levels increased by up to 250% of the secretion in cells exposed to miR-191-5p-enriched EVs compared with controls.

Together, these data demonstrate that the incubation of bronchial epithelial cells with miR-191-5p-enriched EVs results in the upregulation of proinflammatory signaling.

### 3.3. Correlation of EV-Derived miR-191-5p with Clinical Parameters

Table 1 provides a summary of the key clinical characteristics of the patients, which have been previously detailed in a separate publication [18]. Briefly, 35 stable COPD patients were enrolled, with moderate airflow obstruction and a mild reduction in D_L_CO.

**Figure 4:**
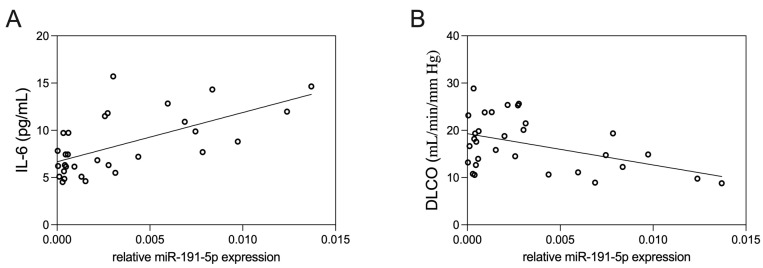
Correlation of EV-derived miR-191-5p and clinical parameters. Correlation analysis was performed to assess the relationships between EV-derived miR-191-5p levels and specific clinical parameters, including IL-6 (**A**) and DLCO (**B**). For technical reasons, IL-6 level was not available in one patient. The analysis was conducted using simple linear regression analysis.

The levels of IL-6 exhibited a mild direct positive correlation with plasma EV-derived miR-191-5p (Figure 4A, *p* = 0.01, *R*^2^ = 0.18). Conversely, we observed an inverse correlation between the levels of EV-derived miR-191-5p and DLCO (Figure 4B, *p* = 0.02, *R*^2^ = 0.15).

### 3.4. Bioinformatic Results

**Figure 5:**
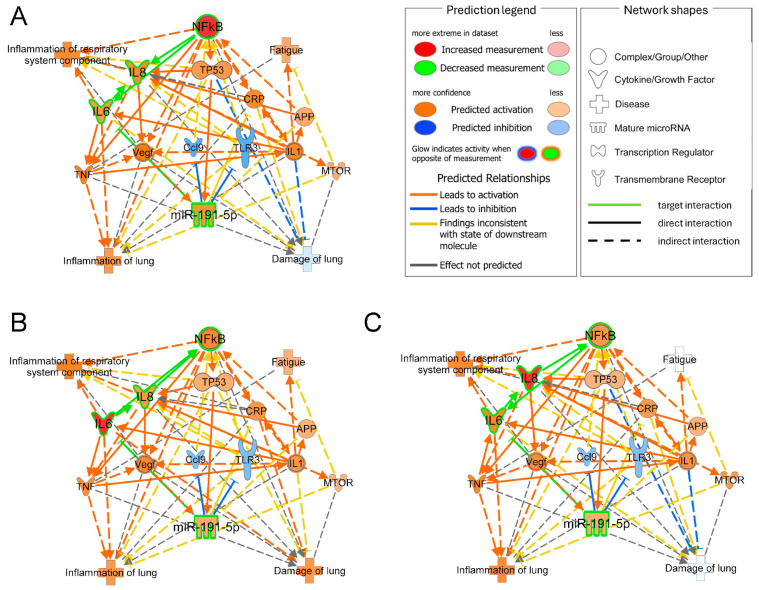
Predictive-network-based analysis involving the activation of NF-κB (**A**), IL-6 (**B**), and IL-8 (**C**). The modulation of the three targets predicted a positive correlation with miR-191-5p, as well as with other nodes related to disease, including IL-1, CRP, and TNF-α, among others. The bioinformatic survey pinpointed a signaling pathway consistent with expression results and reconstructed the molecular neighborhood around targets with several connection nodes that are valuable for a more comprehensive evaluation of the role of miR-191-5p in the pathogenesis of COPD.

Bioinformatic results allowed us to connect target molecules with disease annotations and anticipate potential nodes, enabling us to explain how altered target levels lead to functional impairment. Although the sole upregulation of miR191-5p was not sufficient to predict pathway modulation the individual upregulation of NF-κB, IL-8, and IL-6 resulted in the molecular prediction consistent with the experimental data, exhibiting increased levels of these targets, including miR-191-5p, inflammation, and fatigue (Figure 5A–C). Moreover, this approach allowed us to identify a set of relevant connection nodes such as IL-1, C-reactive protein (CRP), and tumor necrosis factor (TNF-α), representing potential targets for further investigation to gain a deeper understanding of the miR-191-5p network’s role in the pathogenesis of COPD.

## 4. Discussion

This study showed that miR-191-5p-enriched EVs induce proinflammatory effects by upregulating NF-κB activation and IL-8 synthesis in bronchial epithelial cells in vitro. Additionally, we demonstrated a correlation between EV-associated miR-191-5p levels and functional and inflammatory parameters in COPD patients.

In our proposed model, miR-191-5p is transferred from EVs to bronchial epithelial cells, where it exerts proinflammatory effects. Since it is widely recognized that EVs represent the main transporters of circulating miRNAs and the effects of EVs in the recipient cells are dependent on the transfer of functional miRNAs [16], we investigated the impact of miR191-5p-enriched EVs on the in vitro model of bronchial epithelial cells. To this end, bronchial epithelial cells were transfected with miR-191-5p mimic, and we assessed the levels of miR-191-5p in EVs generated following Ach stimulation, a method of EV generation that we have extensively investigated and described in a previous paper [25]. We observed a significant increase in miR-191-5p levels in EVs generated from cells transfected with miRNA mimic, indicating a direct correlation between cellular and EV miRNA levels. Furthermore, we observed that EV-delivered miR-191-5p in bronchial epithelial cells activated NF-kB signaling and increased the synthesis of IL-8. These data are coherent with other studies implicating miR-191-5p in the activation of NF-κB signaling [20]. Moreover, the pathogenic role of IL-8 in COPD, as a chemoattractant for neutrophils in the airways, is well known [32]. In this context, our data corroborate and further expand the proinflammatory effect exerted by EVs, indicating miR-191-5p as one of the cargo actors involved.

In this framework, it is conceivable that bronchial epithelial cells releasing miR-191-5p-enriched EVs modulate the phenotype of distant cells (paracrine effectors) via EV uptake, further propagating the predominantly proinflammatory profiles that characterize patients with COPD [33]. Among the current inhaled treatments for COPD, the muscarinic antagonist tiotropium has demonstrated efficacy in inducing symptom relief and reducing exacerbations, possibly linked to anti-inflammatory effects [34]. We have already demonstrated that tiotropium inhibits the synthesis of IL-8 by bronchial epithelial cells exposed to EVs derived from Ach-stimulated bronchial epithelial cells in an autocrine fashion [25]. Taken together, our findings are consistent with a pathogenic role for both EVs, whose proinflammatory potential is dampened by tiotropium, and EV cargo miR-191-5p, whose paracrine loop with bronchial inflammation should be regarded as an attractive target to attenuate inflammation-associated outcomes in COPD patients.

In our study, we also found that the levels of miR-191-5p, isolated from circulating EVs drawn from stable COPD patients, are correlated with inflammatory (levels of IL-6) and functional (assessment of DLCO) parameters. Persistently elevated circulating levels of IL-6, as a marker of systemic inflammation, are associated with worse outcomes in COPD patients in longitudinal studies [35]. Moreover, in a previous study, we found a moderate positive correlation between monocyte-derived EVs and IL-6 in stable COPD patients [36]. On the other side, DLCO is correlated with the presence and extent of pulmonary emphysema [29] and has a prognostic role in COPD [37]. A negative correlation between CD31^+^ endothelium-derived EVs, markers of endothelial cell apoptosis, and DLCO values has been demonstrated in stable COPD patients, thus suggesting that CD31^+^ EVs could represent a marker of lung parenchyma destruction and emphysema [38]. Serban et al. have also demonstrated the presence of higher amounts of miR-191-5p-containing EVs released from human endothelial cells stimulated by cigarette smoke, thus suggesting a role for miR-191-5p in the inflammatory processes that characterize COPD [22]. Besides demonstrating the presence of miR-191-5p in circulating EVs from COPD patients, our results indicate that miR-191-5p within circulating EVs could be involved in such inflammatory processes. Indeed, EV-associated miR-191-5p levels can be related to both systemic inflammation (expressed by IL-6) and pulmonary damage (estimated through DLCO values), which can be reasonably considered the result of local inflammation. This conclusion is consistent with the in vitro model we have proposed in this study. Of course, the small sample size and the cross-sectional design of the clinical study significantly limited the strength of our results, and we cannot derive conclusions on the possible role of miR-191-5p as a pathogenic player in vivo in COPD patients. Moreover, we did not identify the cell origin of EVs measured in the plasma of COPD patients, even though we recognize that this information could be helpful to better elucidate the mechanisms of bronchial and systemic inflammation characterizing the disease.

**Figure 6:**
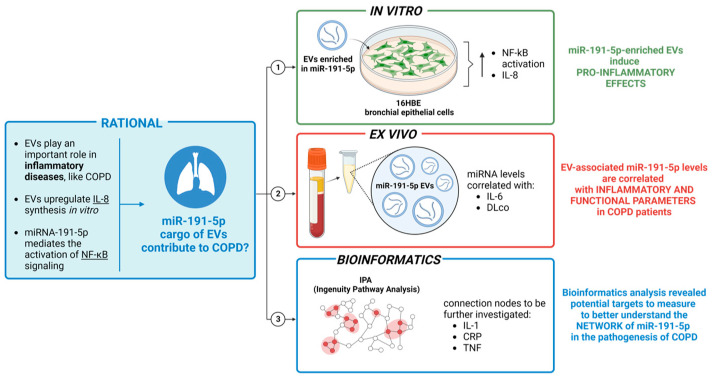
Overview of the study main findings. The figure was created through “biorender.com” website license agreement number HP2679WJTA, access date 11 December 2024.

In summary, this study underscores the critical involvement of EV cargo miR-191-5p in the regulation of inflammation. As depicted in Figure 6, EV-miR-191-5p activates the NF-kB signaling pathway, resulting in elevated levels of IL-8. Interestingly, our bioinformatic analysis has revealed pertinent connecting nodes such as IL-1, CRP, and TNF, which merit further investigation to gain a deeper understanding of miR-191-5p’s role in COPD pathogenesis.

## 5. Conclusions

The present data might help recognize a novel pathogenetic mechanism of COPD that is potentially amenable to therapeutic modulation. Indeed, since the role of EVs in clinically relevant pathways is largely recognized, the evaluation of molecules that interfere with EV genesis and activity is an active field of research [39,40,41].

## Figures and Tables

**Table 1:** Clinical characteristics of the patients.

Age, years	71.0 (6.0)
Birth sex, female:male, n	9:26
Smoking history	
- current smokers, n	9
- former smokers, n	26
Pack-years	49.5 ± 23.4
Dyspnea, mMRC	1 (1)
BMI, Kg/m^2^	29.6 ± 6.1
FEV1, L	1.34 ± 0.44
% pred.	53.9 ± 11.8
FEV1/FVC, %	48.0 ± 11.3
TLC, L	6.70 ± 1.49
% pred.	112.0 ± 19.3
DLCO, mL/min/mmHg	17.1 ± 5.5
% pred.	72.8 ± 22.5

mMRC: modified Medical Research Council; BMI: body mass index; FEV1: forced expiratory volume in 1 s; FVC: forced vital capacity; TLC: total lung capacity; DLCO: diffusing capacity of the lung for CO. Data are reported as mean ± standard deviation or median (interquartile range) as appropriate (Shapiro–Wilk test for normality).

## Data Availability

The dataset of experiments has been deposited to Zenodo’s open-access archive in order to preserve the research results (https://doi.org/10.5281/zenodo.10141977 (accessed on 7 July 2024)).
